# The burden of road traffic injuries in the northeast of Iran: the result of a population-based registry

**DOI:** 10.5249/jivr.v12i1.1265

**Published:** 2020-01

**Authors:** Seyed Reza Khatibi, Hossein Dinpanah, Khadije Maajani, Mahmoud Khodadost, Behnam khodadost, Samaneh Kakhki, Nader Mahdavi

**Affiliations:** ^*a*^ Department of Epidemiology, School of Public Health, Iran University of Medical Sciences, Tehran, Islamic Republic of Iran.; ^*b*^ Department of Public Health, School of Health, Torbat Heydariyeh University of Medical Sciences, Torbat Heydariyeh, Iran.; ^*c*^ Department of Emergency Medicine, 9-Day Hospital, Torbat Heydariyeh University of Medical Sciences, Torbat Heydariyeh, Iran.; ^*d*^ Health Sciences Research Center, Torbat Heydariyeh University of Medical Sciences, Torbat Heydariyeh, Iran.; ^*e*^ Department of Epidemiology and Biostatistics, School of Public Health, Tehran University of Medical Sciences, Tehran, Islamic Re-public of Iran.; ^*f*^ Department of Epidemiology, School of Public Health, Shahid Beheshti University of Medical Sciences, Tehran, Islamic Republic of Iran.; ^*g*^ Department of Nursing, School of Nursing and Midwifery, Torbat Heydariyeh University of Medical Sciences, Torbat Heydariyeh, Iran.

**Keywords:** Burden of disease, Road traffic injuries, Disability-adjusted life years, YLL, YLD

## Abstract

**Background:**

Road traffic injuries (RTIs) are an important public health problem around the world, with the majority of RTIs occurring in low- and middle-income countries. This study aimed to determine disability-adjusted life years (DALY) of RTIs in the northeast of Iran.

**Methods:**

In this cross-sectional study, we used the death registration system to calculate years of life lost (YLL) due to RTIs. To determine the years lost due to disability (YLD), hospital records of all peo-ple injured in road accidents were used. To estimate DALY, we used YLD and YLL to calculate DALY according to the Global Burden of Disease (GBD) 2003 guideline and the age/sex composition of the population was taken from the Statistical Centre of Iran (SCI) in 2016. All collected data entered into Excel software and performed calculations.

**Results:**

Our findings showed that a total of 3403 RTIs and 132 deaths were recorded in 2016. The DALY was 38 per 1,000 of which 26.9 per 1,000 were related to YLL and 11.1 per 1,000 were related to YLD. The highest YLL rate in both sexes was in the 15-29 and 30-44 age groups with 49.8 per 1,000 and 46.0 per 1,000, respectively. This reflects a sex ratio of DALY in males (57.7 per 1,000) to females (8.6 per 1,000) was 6.7.

**Conclusions:**

It seems to be necessary, appropriate effective intervention programs and periodic evaluations are required regarding prevention and reducing traffic accidents mostly in middle-aged men.

## Introdction

Road traffic injuries are one of the most important health problems and the ninth leading cause of death in the world.^1,2^ The burden of road traffic injuries in the community of Iran is very high, as the total amount of years of life lost due to road traffic injuries are more than other causes of death.^3,4^ Every year, approximately 1.35 million people are killed and between 20 and 50 million people are injured by road traffic accidents worldwide and according to the global burden of disease study 2010, RTIs accounted for more than a third of global injury burden, which resulted in the loss of 76 million disability-adjusted life years (DALYs).^5^ It is predicted that with further economic development and increased motorization, global road traffic deaths will increase by more than 35 % from 2010 to 2020.^6^ Also, without sustained action, RTIs will become the seventh leading cause of death by 2030.^2,7^

The situation of road traffic injuries between the different regions of the world is completely different. Approximately 90% of these RTI deaths are concentrated in low- and middle-income countries (LMICs).^2^ Iran is located in the region (Eastern Mediterranean) where traffic-related death rates are the second-highest in the world.^8^ Despite the latest World Health Organization (WHO) report, Iran’s road traffic deaths fell from 32.1 per 100,000 population in 2015 to 20.5 in 2018,^5^ but this is above the global average. According to the previous studies, RTIs are the second cause of death, the first leading cause of years of life lost due to premature mortality (YLL), the second leading cause of disability-adjusted life years (DALYs) after cardiovascular diseases, and the most common cause of injury in Iran.^8-11^ Studies also have shown that in Iran, 13.5 percent of life lost is due to RTIs, which has a high rate compared to the world and the Eastern Mediterranean region.^12-14^

According to the report published by the Legal Medicine Organization of Iran, in the years 2016 and 2017, 15932 and 16201 cases of death and 333071 and 335995 injured cases occurred due to RTIs in Iran, respectively.^15^ More than 50% of deaths from RTIs occur among people aged 15–44 years, which are considered the economically active population in the community.^12,16^ Therefore, the negative impact of these deaths on the life expectancy at birth, and consequently on the economy and society, especially in the health care sector of the country, will be inevitable. ^17,18^ In this regard, it is essential to have accurate and reliable information on deaths and injuries due to road traffic accidents(RTAs) since it would be a clue for several purposes such as monitoring the current status and trends in incidence and mortality rates over time, developing preventive activities, evaluating the progress of road safety management programs, and comparing the RTIs burden to other causes. The burden of diseases also mainly reported the international and national level and estimated, it is recommended to calculate the disease burden based on the sub-national or local level^19^ and registry-based data. The northeast of Iran is vulnerable for RTIs due to its geographical location and high volume of passengers. In order to consider the importance of the different consequences of RTIs on the community, calculate the proper estimates of the burden of injuries based on local and registry-based data, and to determine sub-national and local priorities for decision-makers, we need for further studies to properly understand the epidemiology of RTIs. Therefore, this study was designed to determine the burden of injuries caused by road traffic injuries in the northeast of Iran.

## Materials and Methods

The present burden of disease study was carried out on all the victims of road traffic accidents (fatal and nonfatal injuries), which took place over a period of one year (21st March 2016 to 20th March 2017) on urban and rural roads of Torbat-e-Heidarieh. In order to collect all hospital morbidities regarding RTIs data for YLD, we used the structured questionnaire by referring to the electronic traffic injury information system and hospital records in emergency registration of Nohom-Dey and Razi educational hospitals of Torbat-e-Heidarieh. All admissions to emergency departments in Torbat-e-Heidarieh city in 2016 with the origin of RTIs (V01-V99) were collected. The database consisted of demographic information (full name, age, gender, region), type of vehicle (pedestrian, bicycle, motorcycle, and car), place of crashes, date, treatment outcome, final diagnoses and all external causes based on the International Classification of Diseases (ICD 10) plus cost attributed to such conditions. It is necessary to explain that, from 2005, according to the executive guidelines of Article 92 of the Fourth Economic, Social and Cultural Development Plan in the country, all public, governmental and private hospitals are compelled to admit all inpatients and outpatients who have been injured due to RTAs and provide free-of-charge treatment for injuries. The cost of providing these therapeutic services is funded by using 10 percent of third party car insurance that was deposited into the special income account belong to the Ministry of Health and Medical Education. ^20^ The required data were collected by a trained group of physicians and nurses. The collected information by hospitals was submitted to the deputy of health from all provinces for each year. Also, in this study, data on the deaths due to RTIs were collected from the death registration system of the province health center. Mortality data for the death registration system was collected based on the standard death certificate forms from all sources, such as hospitals, cemeteries, forensic medicine organization, and health centers. The main cause of death reported according to the ICD10 classification was determined by the death certificate. Road traffic deaths have defined all deaths that occur during a road traffic crash or within 30 days after the accident due to road traffic injuries.1 To calculate the incidence rate (age-standardized mortality and non-fatal incidence rate) due to RTIs in 2016, for denominators and populations at risk, the demographic information (the age/sex composition) of the population in 2016 was taken from the Statistical Centre of Iran (SCI). 

In order to calculate the burden of disease, it is first necessary, to sum up, the number of years of life lost due to RTIs (YLL) and the number of years lost due to disability (YLD). Finally, we estimate the DALY, which represents the number of years of life lost due to early death and years lived with the disability of specified intensity and duration. In this study, we used the method of the Global Burden of Disease (GBD) study in 2003 to estimate the burden of road traffic injuries based on DALY.^21^ To estimate the years of life lost due to premature death, we used the standard expected years of life lost (SEYLL). We used the standard life expectancy at each age to estimate the years of life lost due to death at that age. We estimate standard life expectancy based on a specific life table. 

The biological difference in survival between men and women is about 2.5 years and the Coale and Demeny (Model West, level 26) life table model for men and women were used to determine the life expectancy of different age groups. Using the patterns of the table with the Excel framework, which is available from the main designers of the project of burden of disease, and using the burden of disease project weights in the year of 2000, which are consistent with the views of the Iranian experts in the social medicine of the Shiraz University of Medical Sciences, the YLLs, YLDs and DALYs are subjected to the calculations.

YLL(3.1)=N Ce(ra)/(β+r)2[e-(β+r)(L+a)[-(β+r)(L+a)-1]-e-(β+r)a[-(β+r)a-1]]

In this regard

N: The number of deaths in a given age and sex

L: The standard life expectancy of the dead people at the same age and sex

R: Discount rate that’s equal to 0.03

β: The contractual rate is based on the age value of 0.04

C: constant number 0.1658

YLD (3.1) =I*DW*Ce(ra)/(β+r)2[e-(β+r)(L+a)[-(β+r)(L+a)-1]-e-(β+r)a[-(β+r)a-1]]

In this regard too:

1. The number of new morbidity of a disease or injury over a given time

L. Duration of the disease or complication

DW- the weight of a disease or complication

R: Discount rate that’s equal to 0.03

β- The contractual rate is based on the age value of 0.04

C- Constant number 0.1658

DALY=YLL+YLD

Also, the world (WHO 2000-2025) standard population used to calculate age-standardized (adjusted) incidence rate.^22^

## Results

A total of 132 deaths from 3403 RTIs were included in this study. The mean and standard deviation of the injured cases were 29.6 ± 16.3 years. We found that men had higher incidence rates for RTIs (78.17%) than women. Among those who died from RTIs, 102 (77.3%) of them were men and 30 cases (38.8%) were women. Most cases of RTIs were in the age group of 15-29 years with a relative frequency of 50%.

The mortality rate and age-standardized mortality rate of RTIs were 59.1 and 65.45 per 100000 population in Torbat-e-Heydarieh, respectively. According to the sex, the highest mortality rates were in men to 89.8 per 100000 population, and aged 70-79 years old with 152.9 per 100000 population have the highest mortality rate (Table 1).

**Table 1:** The age and sex-specific mortality rate of road traffic injuries (per 100,000 population) in Torbat-e-Heydariyeh city 2016.

Age groups	Population			Number of deaths			Mortality rate			Age- standardized mortality rate
	Male	Female	Total	Male	Female	Total	Male	Female	Total	
0-4	10174	9879	20053	3	5	8	29.5	50.6	39.9	3.53
5-14	22961	22017	44978	8	2	10	34.8	9.1	22.2	3.84
15-29	37662	35247	72909	31	7	38	82.3	19.9	52.1	12.87
30-44	23129	22446	45575	29	6	35	125.4	26.7	76.8	16.40
45-59	10196	10647	20843	16	5	21	156.9	47.0	100.8	16.09
60-69	4418	4774	9192	7	2	9	158.4	41.9	97.9	6.54
70-79	3431	3109	6540	7	3	10	204.0	96.5	152.9	5.70
+80	1626	1614	3240	1	0	1	61.5	0.0	30.9	.48
Total	113597	109733	223330	102	30	132	89.8	27.3	59.1	65.45

The incidence rate and age-standardized incidence rate of RTIs were 2114.36 and 2109.87 per 100000 population in Torbat-e-Heydarieh, respectively. According to the sex, the highest incidence rate was in men with 3269.45 per 100000 population, and people aged 15–29 years with 796.59 per 100000 population have the highest age-standardized incidence rate (Table 2).

**Table 2:** The age and sex-specific incidence rate of road traffic injuries (per 100,000 population) in Torbat -e-Heydariyeh city 2016.

Age groups	Population			Number of cases (fatal and non-fatal)			incidence rate			age- standardized incidence rate
	Male	Female	Total	Male	Female	Total	Male	Female	Total	
0-4	10174	9879	20053	75	46	121	737.17	465.63	603.40	53.46
5-14	22961	22017	44978	220	125	345	958.15	567.74	767.04	132.62
15-29	37662	35247	72909	2021	338	2359	5366.15	958.95	3235.54	796.59
30-44	23129	22446	45575	694	248	942	3000.56	1104.87	2066.92	441.29
45-59	10196	10647	20843	434	171	605	4256.57	1606.09	2902.65	463.26
60-69	4418	4774	9192	129	43	172	2919.87	900.71	1871.19	124.99
70-79	3431	3109	6540	108	33	141	3147.77	1061.43	2155.96	80.42
+80	1626	1614	3240	33	4	37	2029.52	247.83	1141.98	17.24
Total	113597	109733	223330	3714	1008	4722	3269.45	918.59	2114.36	2109.87

The total of 6007.6 years in the Torbat-e-Heydarieh population was lost due to premature death from RTIs in 2016. The years of life lost (YLL per 1000 population) due to RTIs in both sexes, males and females were 26.9, 39.5 and 13.8 per 1000 population, respectively. The most lost years related to premature death was in 30-44 age group (54.6 per 1000 population), 15-29 age group (46.8 per 1000 population) and 45-59 age group (45.7 per 1000 population) for male, and in 0-4 (40.6 per 1000 population), 45-59 age groups (14.8 per 1000 population), 30-44 age group (13.3 per 1000 population) for female, respectively. So, the most YLL due to premature death was occurring in the age group of 15 to 29 with 34.3 % and 74.7 % were in men. The highest rate of years of life lost (YLL) due to premature death from RTIs was related to people aged 30–44 years with 34.3 per 1000 population (Table 3).

**Table 3:** The years of life lost (YLLs) due to premature death, per 1000 population due to road traffic injuries by age and sex groups in Torbat-e-Heydariyeh city 2016.

Age groups	YLLs* (year)			YLL rate per 1,000 population		
	Male	Female	Total	Male	Female	Total
**0-4**	233.3	401.4	634.7	22.9	40.6	31.7
**5-14**	573.5	151.2	724.7	25.0	6.9	16.1
**15-29**	1762.3	423.4	2185.7	46.8	12.0	30.0
**30-44**	1263.4	299.4	1562.7	54.6	13.3	34.3
**45-59**	466.1	157.9	624.1	45.7	14.8	29.9
**60-69**	120.1	45.1	165.2	27.2	9.4	18.0
**70-79**	67.8	38.8	106.6	19.8	12.5	16.3
**+80**	3.9	0.0	3.9	2.4	0.0	1.2
**Total**	4490.5	1517.1	6007.6	39.5	13.8	26.9

*YLL: years of life lost

The number of years lived with disability due to RTIs in the population was 2470.8 years, of the 58.4% were in people aged 15-29 years, and 83.5% were male (Table 4).

**Table 4:** The years lost due to disability (YLDs) per 1000 cases due to road traffic injuries by age and sex groups in Torbat-e-Heydariyeh city 2016.

Age groups	YLDs** (year)			YLL rate per 1,000 population		
	Male	Female	Total	Male	Female	Total
**0-4**	43.8	22.0	65.8	4.3	2.2	3.3
**5-14**	158.0	23.6	181.5	6.9	1.1	4.0
**15-29**	1213.9	228.2	1442.1	32.2	6.5	19.8
**30-44**	454.0	80.3	534.3	19.6	3.6	11.7
**45-59**	125.8	41.8	167.6	12.3	3.9	8.0
**60-69**	43.3	7.8	51.0	9.8	1.6	5.6
**70-79**	15.2	5.0	20.2	4.4	1.6	3.1
**+80**	8.1	0.0	8.1	5.0	0.0	2.5
**Total**	2062.1	408.7	2470.8	18.2	3.7	11.1

** YLD: Years lost due to disability

According to Table 5, the number of disability-adjusted life years (DALYs) was 8478.4, of the 42.8% were in the age group of 15-29 years, and 77.3% of these were in men. The DALY due to RTIs in Torbat-e-Heydarieh was 38 per 1000 population and in the age group of 15-29 years, DALY had a higher rate with 49.8 per 1000 population. The highest YLDs according to the injuries were Long-term hip fracture (727.2), spinal cord injury (694.3), and long-term skull injury (691.4), respectively.

**Table 5:** Distribution of YLLs, YLDs, DALYs and DALY in 1000 population due to road traffic injuries by age and sex groups in Torbat-e-Heydariyeh city 2016.

Age groups	Male		Female		Male and Female	
	DALYs†	DALY per 1,000 population	DALYs	DALY per 1,000 population	DALYs	DALY per 1,000 population
**0-4**	277.1	27.2	423.4	42.9	700.6	34.9
**5-14**	731.5	31.9	174.7	7.9	906.2	20.1
**15-29**	2976.2	79.0	651.5	18.5	3627.8	49.8
**30-44**	1717.4	74.3	379.7	16.9	2097.1	46.0
**45-59**	591.9	58.1	199.8	18.8	791.7	38.0
**60-69**	163.4	37.0	52.9	11.1	216.2	23.5
**70-79**	83.0	24.2	43.8	14.1	126.8	19.4
**+80**	12.0	7.4	0.0	0.0	12.0	3.7
**Total**	6552.5	57.7	1925.8	8.6	8478.4	38.0

†DALYs: disability-adjusted life years

Furthermore, the rates of YLL, YLD, and DALYs were found to be higher in males than in females, with 39.5, 18.2 and 57.7 per 1000 population, respectively. The highest YLL was in the age group of 30-45 year (34.3 per 1000 population), YLD was in the age group of 15-9 years (19.8 per 1000 population), and DALYs were in the age group of 15-29 years (49.8 per 1000 population).

The number of disability-adjusted life years in the Torbat-e-Heydarieh in 2016 was 38 per 1000 population, of which 26.9 per 1000 population were due to early death and 11.1 per 1,000 of them were lost due to disability. The two age groups of 15-29 and 30-44, with 49.8 and 46.0 per 1000 population had the highest number of years of life lost due to premature death and disability. Men with a rate of 57.7 per 1000 population had a higher rate in DALY than women (8.6 per 1000 population).

## Discussion

The findings set out here highlight the high rates of the mortality rate (59.1 per 100000 populations) and the age-standardized mortality rate (62.7 per 100000 population) due to RTIs in the northeast of Iran which is more than the average mortality rate of Iran and provinces level (20.5 in Iran, 21.1 in Isfahan, 28.8 in Khuzestan, 33.0 in Tehran, 33.3 in Northern provinces, 33.3 in Lorestan, 46.4 in Yazd, and 51.3 per 100000 population in Kermanshah provinces), and other regions in the world (17.4 in the World, 19.9 in Eastern Mediterranean Region, 9.3 in Europe, 26.6 per 100000 population in Africa) in other recent studies.^5,23-29^

This finding showed that the mortality rate due to RTIs was 3.1 times higher than in Iran rate and 3.6 times higher than the world average. The high mortality rate, compared to other communities may be attributed to many factors such as the strategic location, age structure of the populations, types of vehicles, quality and safety of vehicles, different traveling patterns or may be rooted in risky driving behaviors in this community. Therefore, further studies are required to determine factors affecting accidents and mortality rates due to RTIs and implement appropriate intervention programs for intensified safety promotion work.

Our study showed that the male to female (M/F) sex ratio for mortality rate in the present study was almost 3:1 (89.8 versus 27.3 per 100000 population) that are in line with the results of studies in other provinces of Iran.^24,26,28^ Also, the majority of RTIs (78.5%) in the northeast of Iran occurred in men, which is similar to the findings of other studies in Iran, other countries, and worldwide.^30-33,5^ Thus, men were approximately four times more likely injured or killed due to RTAs compared to women. In Thailand, ^34,35^ however, that number was up to five times.^34,35^

The present study, consistent with other studies in Iran^24,26,28^ revealed that the age group most greatly affected by RTIs was the age group 15-44 years (70.0%), but the mortality rate was increasing with age, also the highest rate in both sexes was among the age group 70-79 years despite their lower incidence rate. However, the results of Ayatollahi et al.^36^ and Montazeri et al.^37^ showed that the highest mortality rate was concentrated in younger age groups and decreased with age. The pattern of high mortality rate due to RTIs among the elderly may be rooted in their decreased mobility (especially for pedestrians) and higher vulnerability due to the underlying problems, so this age group has a double chance of dying.^38,39^ These gender differences and suffering three times more from road injuries in men than women^40,41^ may be due to the greater role in transport and driving, ^42^ and the higher prevalence of risky behaviors in men. It seems also that men would be involved in more serious crashes. So it can be the main reason to prioritize the development of a sex-oriented driving education program.

This study has shown that the age-standardized incidence rate of RTIs (fatal and non-fatal) was 2109.87 per 100000 population in the Torbat-e-Heydarieh, which is more than the incidence rate of RTIs for Iran (405.0 per 100,000 population) in Shavaleh et al. study.^43^ This number is only lower than the number of road traffic injury rates in Nigeria (4120.0 per 100 000 population). ^44^ In the present study, also the age group of 15-29 years has the highest incidence rate (796.59 per 100000 population) compared to other age groups. According to the results, young drivers were considerably more likely to engage in road traffic accidents (RTAs) mainly due to inadequate experience as well as risky behaviors due to peer relationships.^45,46^ To implement an effective policy to reduce RTIs, it is essential to identify and classify different groups of people and separate vulnerable road users from other road users.^47^

This study has shown that, the disability-adjusted life years (DALYs) due to RTIs in both sexes was 38.0 per 1000 population (8478.4 years) and from this value, 26.9 per 1000 population (6007.6 years) was attributed to lost years related to premature death (YLL) and 11.1 per 1000 population (2470.8 years) was attributed to years lost due to disability (YLD). These results revealed the greater value for these rates compared to other studies.^12,23,34,36,48^ In the present study, 70.8% and 29.2% of the total DALY were related to YLL and YLD, respectively. Similar results have been reported from other studies in Iran (64%). Isfahan (83%), Yazd (87.4 %)and South Khorasan (95.0%) provinces,^12,23,28,36^ and in other countries in Thailand (88%), France 65%, Swiss canton (70%), Australia (73%),and Serbia (57%).^34,49-52^ Nevertheless, in Isfahan province of Iran (83%)^23^ and the first study of the burden of disease and injury in Iran 2003 which, showed that 70%, 67%, and 66% of total DALY in Hormozghan and Khorasan, East Azerbaijan, Yazd, and Chaharmahal-Bakhtiari provinces of Iran were related to YLD, respectively.^53^ Such a difference might be due to the deaths that occurred among older people lead to a decrease in the calculated YLL and an increase in YLD. 

So considering that in the present study, the mean age of the victims was 29.6 years and maximum rate of YLL and YLD were in the 15-44 young years-old (69.9% of injuries and deaths were in this age group) and since those are the active groups of the community, therefore, their death or disability places a huge social and economic burden on the community, especially in developing countries. Furthermore, since younger ages due to the high use of vehicles, especially with lower safety, such as motorcycle^54^ are 34-fold increased risk of death and 8 times more likely to be injured for drivers of these vehicles compared to people driving other types of motor vehicles.^55^ Although the risky behviors while driving and inattention to traffic laws^56^ as well as the lack of clear lines between two-wheeled vehicles and four-wheeled vehicles on roads,^28^ Not wearing a helmet, alcohol and drug use, inexperience and driver training, licensure and ownership, and riding speed^57^ have recently been identified as contributing to this risk.

In this study, femoral long-term fracture (29.4%), spinal cord damage (28%) and long-term skull damages (27.9%) respectively, were the most frequent injuries leading to hospitalization which caused YLD in both genders. This finding seems to be consistent with a study conducted in Iran^43^ and another study in France.^49^ But, this finding was inconsistent with previous studies in Iran. The study was done in Isfahan province, the most injuries have been occurred among total injuries in both sexes have been patella, fibula and tibia fractures (21.5%), Intracranial damages (12.8%) and fracture of radius or ulna (8.4%), respectively.^23^ Also in the Kermanshah province of Iran, the most amount of YLD in men was belong to patella, fibula and tibia fracture and in women was belong to the clavicle, shoulder, arm and skull fracture. The most YLD in injuries leading to hospitalization were also related to the sternum, rib and face fracture in both sexes.^29^ In Iran, the majority of RTIs by type of road users occur in Motorcyclist (39.2%), followed by car occupants (28.9%), and pedestrians (20.0%). ^43^ The proper helmet use reduces the risk of head injuries by around 69% and fatal injuries by around 42% for motorcyclists involved in accidents, also the seat-belt usage reduces the risk of death among drivers and front-seat occupants by 45-50%, and the risk of death and serious injuries among rear seat occupants by 25%. ^58^ Therefore, implementing interventions such as safety crossing on roads for pedestrians and enforcement of laws on the use of helmet, seat-belt, and child restraint is essential to reduce deaths and serious injuries in communities. 

In the present study, the burden of RTIs in men is about 6.7 times higher than that of women, which confirmed the highlight gender differences result (3-5 times higher in men) in previous studies in Iran and other countries.^23,34,35,39,53^ Men are more at risk than women because of their job duties and make money and also men tend to drive and Perform high-risk behaviors such as unauthorized speed, inappropriate use of vehicles, crossing unauthorized regions, insufficient use of safety equipment can be named. ^59^

## Conclusion

On the whole, disability-adjusted life years (DALY) caused by RTIs in the Torbate-e-Heydariyeh city impose a high burden on the community. According to its pattern in age and sex groups, especially men in the age group of 15-44, it should be considered as a serious public health problem. 

Therefore, it is recommended, in order to prevent and minimize the occurrence of road accidents, to implement appropriate intervention programs in the community, especially the vulnerable persons aged 15-44 years, including revise laws on the use of motorcycles (especially on helmet use for motorcyclists), enforce strict laws, and periodic evaluation of executive programs at different levels seems necessary. Further studies should examine the factors influencing the occurrence of traffic accidents (especially social determinants). The current findings, combined with future studies will be beneficial for policy-makers for the prevention of RTIs.

**Acknowledgment**

The authors wish to acknowledge the Deputy of Research of Torbat-e-Heydariyeh University of Medical Sciences for approval and financial support of this research project, as well as the emergency room staff's Nohom-Dey and Razi educational hospital of Torbat-e-Heidarieh.
